# Dynamic Thermal Voltage Adaptation for LED Branches in Automotive Applications

**DOI:** 10.3390/s25175392

**Published:** 2025-09-01

**Authors:** Jose R. Martínez-Pérez, Miguel A. Carvajal, Juan J. Santaella, Pablo Escobedo, Nuria López-Ruiz, Antonio Martínez-Olmos

**Affiliations:** 1 R&D Department, Valeo, 23600 Martos, Spain; jose-ramon.martinez-perez@valeo.com (J.R.M.-P.); juan-jose.santaella@valeo.com (J.J.S.); 2 Department of Electronics and Computer Technology, ETSIIT, University of Granada, 18014 Granada, Spain; carvajal@ugr.es (M.A.C.); pabloescobedo@ugr.es (P.E.); nurilr@ugr.es (N.L.-R.)

**Keywords:** automotive lighting, LED, temperature, thermistor, power

## Abstract

This paper presents a novel technique for thermally compensating the power output of a DC-DC converter that supplies automotive lighting/signaling systems with multiple LED branches. The method ensures stable bias voltage for the current drivers controlling each branch, maintaining consistent power consumption across a wide temperature range. This issue has been minimally addressed in existing literature, providing few solutions which are too complex for industrial production. The approach proposed is simple and involves incorporating a temperature-sensitive thermistor into the DC-DC converter’s control loop, enabling the output voltage to adjust with ambient temperature. Different control loop configurations are explored, demonstrating that a simple resistor-thermistor network can approximate the desired voltage response under diverse thermal conditions. The power dissipated in the current drivers is kept within a controlled range, improving system efficiency and reducing heat loss. Additionally, it minimizes the need for additional current drivers, lowering the cost of these systems, improving battery life of the DC-DC converter, and decreasing CO_2_ emissions. For the case studies analyzed, an optimized configuration with appropriate resistor values and thermistor models achieves a 75% relative reduction in power dissipation by the current driver and a 50% improvement in the relative efficiency of the LED branch system.

## 1. Introduction

Electrical lighting and signaling systems in the automotive sector have been widely used since their introduction in 1908, when the first incandescent lamps based on tungsten filament bulbs were implemented [1]. Currently, after more than a century of evolution, the dominant technology for designing lighting and signaling systems in vehicles is solid-state (SS) lighting technology, which emerged in the early 21st century [2]. The use of light-emitting diodes (LEDs) in automotive applications now accounts for 95% of all lighting applications. SS lighting technology offers significant advantages over other types of lamps, such as high brightness, reliability, low power consumption, and long lifespan [3]. Moreover, it provides higher efficiency than previous lighting systems, which is understood as the number of lumens emitted per watt of applied power. White LEDs can achieve efficiencies exceeding 100 lm/W, reaching up to 150 lm/W [4], whereas halogen bulb-based systems are limited to values between 20–25 lm/W [5].

However, SS lighting systems convert only a limited percentage of the supplied energy into light (approximately 30%), while the remainder is converted into heat [6]. This heat generation leads to an increase in device temperature during operation, which results in reduced efficiency due to the high thermal dependence of SS devices and a potential reduction in their lifespan [7]. Ambient temperature also has a strong influence on LED performance. In automotive applications, ambient temperature typically ranges from −40 °C to 105 °C; thus, a maximum operating temperature of 120 °C (junction temperature) is considered for LEDs. Because of these effects, the LED luminous flux is highly temperature-dependent. To manage heat dissipation generated during the operation of SS lamps, numerous techniques have been proposed in recent years. These strategies include junction temperature prediction studies using thermal modelling of both the device and the electronic components required for the current driver implementation [8,9], heat dissipation techniques to maintain a constant internal temperature within the lamp [10,11], and cooling strategies to reduce the lighting system’s temperature [12,13]. Other efforts have focused on compensating for the drift in the luminous flux emitted by SS lamps due to temperature variations, rather than directly correcting the temperature itself [14,15,16]. However, little work has been published on this subject in the existing literature. Among the proposed strategies aimed at stabilizing luminous flux by compensating for temperature effects, a common approach is the use of photodetectors to measure the luminous intensity emitted by the SS light source and microcontrollers to regulate the LED biasing current, thereby correcting the light intensity drift caused by temperature fluctuations [17,18,19]. However, this approach requires substantial modifications to the design of SS lamps typically used in automobile production, which limits its industrial implementation due to the increased complexity and cost of the lighting system.

In contrast, LED branches in SS lamps are typically biased at a constant voltage through DC/DC converters [20,21]. The latest cutting-edge trends in automotive lighting design are increasingly focusing on signaling modules due to their ability to project a distinctive signature that can be associated with the vehicle manufacturer. Illuminated grilles, which incorporate multiple LEDs as light sources, are central to these complex signaling modules, which are becoming a defining feature of automotive manufacturers and a means of communication with pedestrians, other drivers, and autonomous vehicles [22,23]. This new approach to signaling modules has led to novel architectures, primarily involving voltage sources controlling parallel LED branches that are biased by a current controller [24,25,26].

Temperature significantly influences the voltage drop across LEDs, and this voltage decreases with increasing temperature [26]. Consequently, the DC/DC converters responsible for generating voltage for LED branches must be designed to produce the maximum voltage required by the LEDs within each branch of the lamp, corresponding to the lowest operational temperature. However, as the ambient temperature rises, the LED voltage drop also decreases, even at the same current demand. This leads to excess power that is not consumed by the LEDs, resulting in increased energy inefficiency as temperature rises. Moreover, a sharp change in the operating temperature could even compromise the operation of LED fixtures, due to a significant shift in the dynamic resistance of multiple series-connected LEDs [27]. Additionally, if the LED current is regulated—i.e., kept constant—an increase in temperature will lead to a decrease in the LED light output [28]. The available literature presents design solutions aimed at compensating for the thermal dependence of LED voltage drops in lamps to mitigate these effects [29,30,31]. However, similar to existing thermal compensation proposals for LED luminous flux, the proposed solutions involve complex designs that include feedback loops, cooling systems, and advanced control mechanisms. This complexity significantly limits their practical industrial implementation.

In a previous study, the authors presented a luminous flux stabilization system for SS automotive lamps, which reduced the complexity of previous proposals [32]. This study proposed the use of a thermistor as a temperature sensor, acting as a shunt resistor in an integrated current driver. The introduction of the thermistor successfully maintained a stable luminous flux despite temperature variations.

In the present study, the use of a thermistor is again proposed as part of the lighting module design, with the specific objective of regulating the voltage generated by a DC-DC converter to power LED branches in response to temperature fluctuations. This ensures that the voltage applied to the current drivers—which bias the LED branches—remains stable at a minimum level. As a result, the power consumed by these current drivers remains constant, preventing unnecessary energy waste in the form of heat dissipation. Thanks to the inclusion of this passive component, the module required for LED branch biasing in complex lighting grilles can be implemented using commercial DC-DC converters and current drivers, with no additional modifications other than incorporating the thermistor as a temperature-sensitive element. In the following sections, it is demonstrated that the proposed approach can adapt the voltage generated by the DC-DC converter according to the LED requirements of each branch as a function of temperature, while maintaining a constant biasing current.

## 2. Technical Background

**Figure 1:**
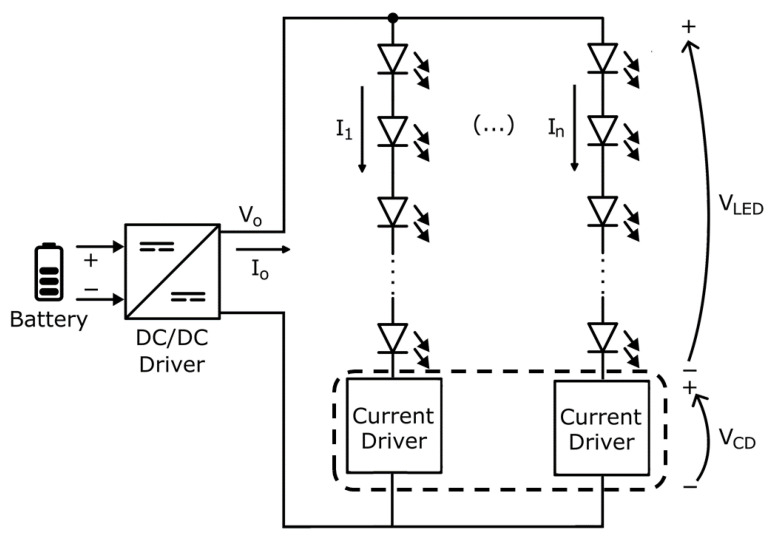
Basic schematic of a lighting module.

The current design trend for solid-state lighting in automotive applications, both for front and rear lights, is based on using a high number of LEDs distributed across multiple branches. These branches can be independently switched on or off to create lighting patterns or signatures associated with each automobile manufacturer. A general schematic of a lighting module in these lamps is depicted in Figure 1.

In this schematic, the DC/DC converter draws power from the vehicle battery and generates a constant voltage *V_o_* and an output current *I_o_* to bias the *n* LED branches that make up the module. This current is distributed among the branches according to the current driver configuration, which forces the current in each branch as follows:

(1) $$I_{o}=\sum_{i=1}^{n}I_{i}$$

The power generated by the DC-DC converter and delivered to the lighting branches *P_o_* is given by:

(2) $$P_{o}=V_{o}\times I_{o}$$

The generated voltage *V_o_* is distributed between the voltage drop required by each LED branch—which are assumed to be equal in this case—(*V_LEDs_*), and the voltage drop in the integrated current driver shown in Figure 1 (*V_CD_*):

(3) $$V_{o}=V_{LEDs}+V_{CD}$$

As previously stated, the voltage drop across LEDs connected in series within a branch is temperature-dependent and decreases with increasing temperature. Therefore, the value of *V_o_* must be calculated to ensure proper circuit biasing in the worst-case scenario, which occurs at the lowest ambient temperature of the application. For automotive lighting, this corresponds to −40 °C, while also considering the minimum voltage required by the current driver to function (approximately 1 V). At higher temperatures, the term *V_LEDs_* decreases, and the excess generated voltage is absorbed by the current driver, leading to increased temperature in this device. In other words, the excess energy received is dissipated as heat.

Although alternative design approaches have been proposed to compensate for the thermal variation of the LED forward voltage, as discussed in the Introduction, their complexity hinders industrial-scale implementation. As a result, the increase in voltage drop across the current driver with temperature is typically accepted, despite causing inefficient energy utilization from the vehicle’s battery. Moreover, overheating in the current driver may necessitate increasing the number of integrated circuits of this type within the lighting modules or expanding the heat dissipation areas.

The objective of this study is to propose a design alternative that, following the schematic in Figure 1, provides a dynamic adaptation of the required voltage *V_o_* based on temperature. This approach ensures that the voltage applied to the current driver remains at a stable minimum level, thereby preventing excessive power consumption—which would otherwise generate unnecessary heat—while maintaining sufficient simplicity to ensure feasibility for industrial-scale production.

## 3. Thermal Model

**Figure 2:**
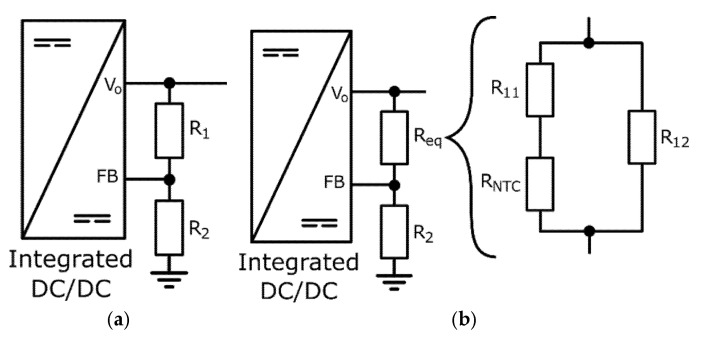
(**a**) Configuration of a DC-DC converter with a resistive feedback loop; (**b**) alternative resistive network to R1.

Modern vehicle lighting and signaling systems consist of LED branch arrays, as shown in Figure 1. These branches are biased by integrated current drivers that maintain a constant biasing current. DC-DC converters used for the polarization of LED branches in automotive lamps include feedback loops that allow, through external passive components such as resistors, adjust the output voltage to the desired value or its adaptation to the load [25]. For the converter scheme shown in Figure 2a, the generated output voltage is given by [33]:

(4) Vo=α1+R1R2

where the parameter *α* is a coefficient specific to the DC-DC converter (*α* ≤ 1). As derived from Equation (4), the output voltage of the converter is a constant value that depends on *R*_1_ and *R*_2_. The forward voltage of an LED (*V_F_*) is temperature-dependent, and this dependence can be modelled, as a first approximation, by a linear relationship, as shown in Equation (5) [34], when the bias current is constant:

(5) $$V_{F}=V_{i}+k_{T}\left(T-T_{i}\right)$$

where *V_i_* and *T_i_* are the reference voltage and temperature of each LED, respectively, *k_T_* is the proportionality constant, and *T* is the current temperature of the LED. In the case of *m* identical LEDs connected in series, the voltage drop across the branch will be:

(6) $$V_{F}(T)=m\cdot \left(V_{i}+k_{T}\left(T-T_{i}\right)\right)$$

At the temperature extremes of the LED-based lighting and signaling system’s operating range, for a branch of m LEDs:

(7) $$\left.\begin{array}{l} V_{F}(T_{\min})=m\cdot \left(V_{i}+k_{T}\left(T_{\min}-T_{i}\right)\right)\\ V_{F}(T_{\max})=m\cdot \left(V_{i}+k_{T}\left(T_{\max}-T_{i}\right)\right) \end{array}\right\}$$

From these expressions, the variation in the voltage drop across an entire branch due to the maximum operating temperature difference can be estimated as:

(8) $$\Delta V_{F}=V_{F}(T_{\min})-V_{F}(T_{\max})=m\cdot k_{T}\left(T_{\min}-T_{\max}\right)$$

This voltage drop *V_F_* corresponds to the term *V_LEDs_* in Equation (3). Therefore, the variation given by Equation (8) represents the maximum overvoltage that the current driver must withstand due to the operating temperature difference of the system. For an integrated DC-DC converter, such as the one shown in Figure 2a, to generate a voltage that thermally adjusts to the voltage demanded by one or more LEDs according to Equation (5), it is necessary to include a temperature-dependent resistive element in the external feedback loop. Such element can be a thermistor, whose resistance varies significantly with ambient temperature. Since the constant *k_T_* is always negative for an LED (its forward voltage decreases with temperature), this work proposes the use of a negative temperature coefficient thermistor (NTC) in the external feedback loop of the DC-DC converter, as shown in Figure 2b, where the resistance *R_1_* of the original voltage divider is replaced by a network of resistors that includes an NTC thermistor. In this way, the equivalent resistance *R_eq_* takes the following expression:

(9) $$R_{eq}=\left(R_{11}+R_{NTC}\right)\left\|R_{12}\right.$$

The resistance of an NTC thermistor is given by:

(10) $$R_{NTC}=R_{0}\cdot e^{B\left(\frac{1}{T}-\frac{1}{T_{0}}\right)}$$

where *R_NTC_* is the resistance at ambient absolute temperature *T*, *R*_0_ is the resistance at temperature *T*_0_, and *B* the constant of the thermistor. From Equations (4), (9) and (10), the output voltage of the DC-DC converter, using the alternative configuration to replace the resistance *R*_1_, can be obtained as follows:

(11) $$V_{o}=\alpha \left(1+\frac{R_{12}\left(R_{11}+R_{0}\cdot e^{B\left(\frac{1}{T}-\frac{1}{T_{0}}\right)}\right)}{R_{2}\left(R_{11}+R_{12}+R_{0}\cdot e^{B\left(\frac{1}{T}-\frac{1}{T_{0}}\right)}\right)}\right)$$

Since this output voltage is used to power the LEDs of the SS lamp, as shown in Figure 1, it must vary approximately linearly with temperature, as described by the expression in Equation (5). As will be demonstrated in the following sections, the proper selection of components for the feedback network of the DC-DC converter shown in Figure 2b will enable this outcome.

## 4. Materials and Methods

**Figure 3:**
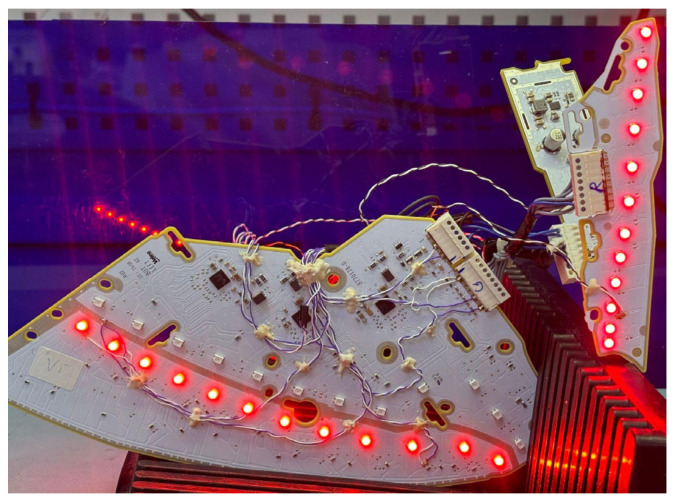
Prototype used for thermal characterization.

To evaluate the proposed thermal model proposed, we designed a lamp consisting of three lighting modules, following the schematic in Figure 1 with the parameters described in Table 1. This design was manufactured on a printed circuit board (PCB), generating three identical systems that served to obtain the replicas of the measurements. The used LED models were DRS-MKS and DRA-HKS (Dominant Opto Technologies, Melaka, Malaysia). The three modules were powered by constant current through three E522.59 current drivers (Elmos Semiconductor, Dortmund, Germany). This device is capable of powering up to 16 branches with a current of up to 100 mA. The voltage applied to all branches of the different modules was obtained through a single DC-DC converter, model MPS MPQ4322GLE (Monolithic Power Systems, Washington, DC, USA). A photograph of the implemented system is shown in Figure 3.

This lamp was evaluated in two scenarios: (i) generating a bias voltage *V_o_* for the LED branches through the DC-DC converter with a classic resistive feedback loop, and (ii) generating the voltage through a loop with a thermistor to compensate for the thermal dependence of the system’s LEDs, as shown in Figure 2b. The power generated by the DC-DC converter was analyzed within the ambient temperature range from −40 °C to 80 °C (beyond which, the thermal protection systems integrated into the lighting modules are activated) by placing the system in a temperature test chamber, model CTS T-70/600 (CTS GmbH, Kall, Germany), which maintains stable temperature conditions in the range from −70 °C to 180 °C. For each temperature setting, a stabilization period of 120 min was allowed before measuring the luminous flux, ensuring a uniform temperature distribution throughout the climatic chamber and the board’s electronic components. The voltage and current measurements of the LEDs in the presented modules were always taken under steady-state conditions. The junction temperature of the LEDs is related to the ambient temperature [32], and the thermistor is placed close to the LEDs to directly estimate this value.

To select the optimal configuration for the DC-DC converter’s feedback loop and the most appropriate values for the resistances and the thermistor, a numerical simulation was conducted to evaluate the voltage generated by the device for different values of the loop parameters and the two proposed configurations. For the LED models considered in this design, as shown in Table 1, the maximum expected voltage drop per LED occurs at −40 °C, with a value of 2.8 V, according to the manufacturer’s data. For a branch of 2 LEDs, and considering that the selected current driver requires 1 V, the required Vo voltage generated by the DC-DC converter is 7.6 V, with an additional 1 V safety margin to ensure proper polarization of the branches (this margin can be reduced in industrial production to avoid oversizing the polarization and minimize energy waste). At the maximum operating temperature in automotive environments, the maximum voltage required for the proposed branches is approximately 6.7 V. Therefore, the feedback loop of the DC-DC converter must be designed to ensure that the generated voltage varies as linearly as possible between these values when the LED junction temperature fluctuates between −40 °C and 120 °C.

To achieve this, various NTC thermistors and resistors were selected to provide the necessary voltage values for *V_o_* in the configuration shown in Figure 2b. Their parameters are listed in Table 2. In this table, configuration #1 corresponds to the static case, where no thermistor is included and the feedback loop follows the classic configuration shown in Figure 2a. On the other hand, configurations #2, #3, and #4 correspond to the dynamic model, which includes a control loop for the complex DC-DC converter, incorporating the thermistor.

## 5. Results

**Figure 4:**
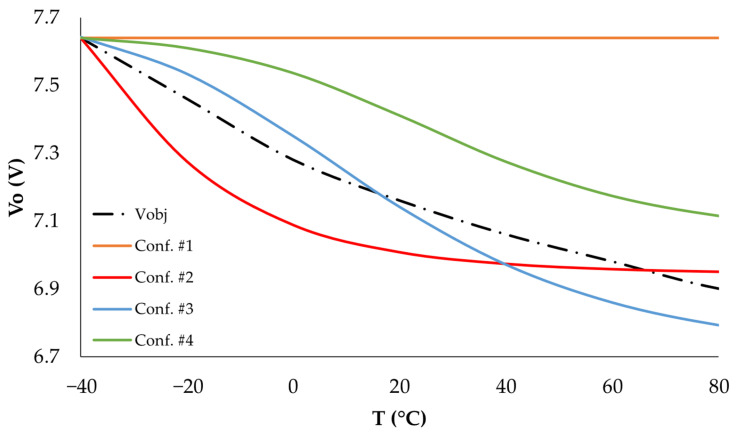
Theoretical variation of the voltage *V_o_* with temperature for different configurations of the DC-DC converter control loop.

Figure 4 presents the theoretical output voltage *V_o_* of the DC-DC converter with the control loop, following the proposed configuration (expressed in Equation (11)), numerically obtained within the temperature range of −40 °C to 80 °C for the values listed in Table 2. This figure also includes an estimation of the variation in the target voltage (*V_obj_*) required by the LED branches, due to changes in the LEDs’ forward voltage within the indicated temperature range, as described by Equation (3).

**Figure 5:**
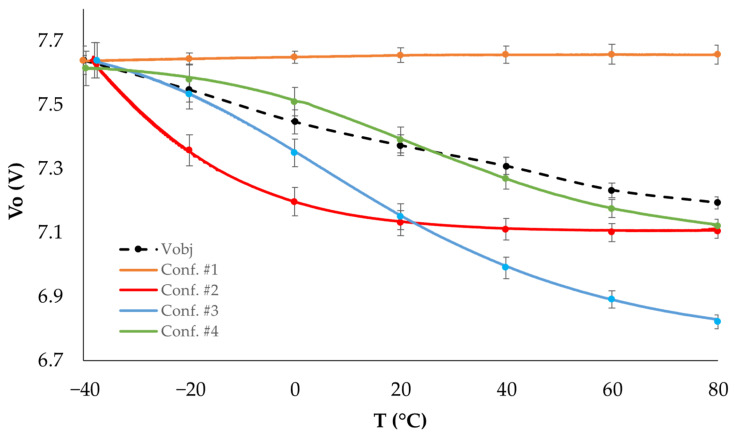
Experimental variation of the voltage *V_o_* with temperature for different configurations of the DC-DC converter control loop.

As shown in Figure 4, the output voltage generated with configurations #2, #3, and #4 from Table 2 varies with temperature, following the expected voltage demand of the LEDs in the lighting system branch. In contrast, configuration #1 (classic feedback without a thermistor) generates a constant output voltage with no thermal dependence. To verify this behavior and select the optimal configuration, the results in Figure 4 were experimentally reproduced using the prototypes shown in Figure 3. The experimental results are presented in Figure 5, displaying the average of the three replicas used for each configuration.

**Figure 6:**
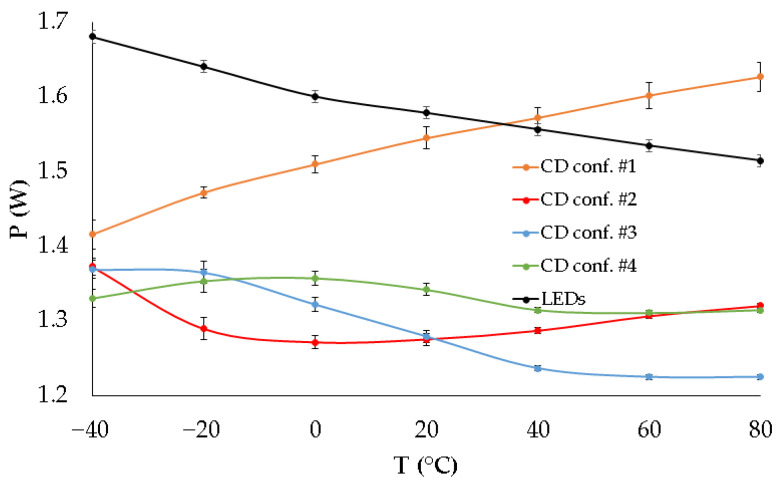
Experimental variation of the power consumed in the current driver (CD) and the LEDs with temperature for different configurations of the DC-DC converter control loop.

The results in Figure 5 show the expected variation of the output voltage with temperature, as predicted by the thermal model from the previous section, for configurations #2, #3, and #4 when the LEDs in each system branch are biased with the currents listed in Table 1. The measured voltage drop in the LED branch slightly deviates from the expected behavior shown in Figure 4, with a lower slope observed in the experimental measurements. This suggests that the feedback loop configuration of the DC-DC converter that generates a voltage closest to the actual voltage demand of the LEDs is configuration #4 from Table 2. For the same configurations, the power dissipated in the current driver of each branch was measured at the current shown in Table 1, with the total bias current for the complete lamp being 390 mA, and the obtained results are shown in Figure 6. As a reference, the power consumed by the LEDs has also been included in this graph. This power is independent of the configuration used in the feedback network of the DC/DC converter.

The results presented in Figure 6 clearly show that the power consumed by the current driver, when the DC-DC control loop does not incorporate thermal adaptation (i.e., consisting only of two resistors as in configuration #1), increases almost linearly with temperature. This behaviour is expected due to the decrease in the LEDs’ forward voltage, as seen in Figure 5. This variation leads to a power increase of approximately 15% within the studied temperature range for this configuration. In contrast, when the feedback loop includes a thermistor to adapt the generated voltage to temperature changes, the power supplied to the current driver remains within a more limited range. Specifically, configuration #4 provides the most stable power consumption in this element, with a maximum variation of 4%, representing a relative reduction of nearly 75% with respect to the classic configuration.

**Figure 7:**
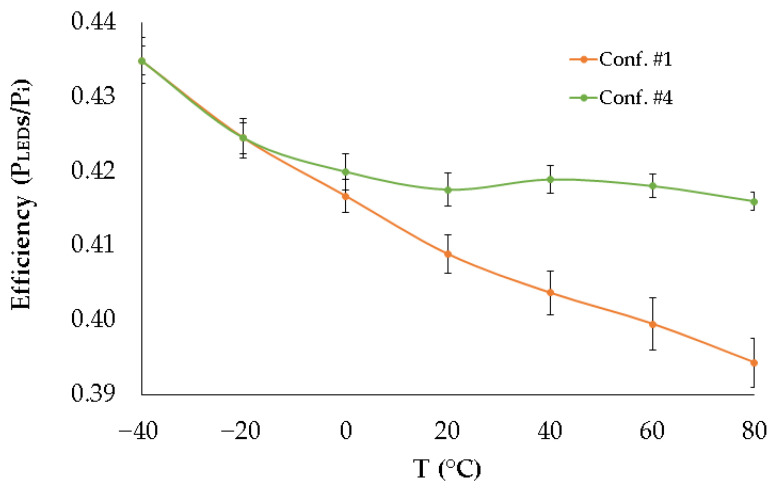
Efficiency of the LED branch biasing system.

As a final performance metric of the proposed thermal adaptation solution in the DC-DC generated voltage, system efficiency was evaluated. Efficiency is defined as the ratio between the power consumed by the LEDs in the branch (*P_LED_*) and the total power delivered to that LED branch (*P_i_*), which also includes the power dissipated in the current driver. Figure 7 presents this efficiency for configurations #1 (classic) and #4 (thermally adapted) of the DC-DC control loop.

The results presented in Figure 7 show that the efficiency of the DC-DC converter in relation to the power supplied to an LED branch remains within a more stable range when the DC-DC control loop follows the topology proposed in this work to thermally adapt its output voltage. In the specific case studied, the absolute efficiency improves by more than 5% using configuration #4 compared to the classic configuration (#1, not thermally adapted) at the maximum analyzed temperature, which represents the worst-case scenario. In terms of relative efficiency (normalized to the maximum value at T = −40 °C), the improvement exceeds 50%.

## 6. Conclusions

In this work, a new technique is presented to thermally compensate for the power generated by a DC-DC converter that supplies an automotive lighting or signaling system composed of multiple LED branches. This approach ensures that the bias voltage of the current driver that activates each branch remains constant, or equivalently, that the power consumed by this driver remains stable. This problem has been rarely explored to date, with few proposed solutions, all of which are not feasible for industrial production because of their design complexity. The proposal in this work is based on a simple solution that incorporates a thermistor, a temperature-sensitive component, into the DC-DC converter’s control loop. This inclusion causes the converter’s output voltage—and consequently the generated power—to adapt to ambient temperature.

Several configurations for the DC-DC control loop were proposed, all of which demonstrated that the generated voltage can be approximated, using a simple network of resistors and a thermistor, to the voltage required by the LEDs in a branch over a wide operating temperature range. The power dissipated in the current driver of each branch remained within a limited range throughout this temperature range thanks to the proposed configuration. This increases system efficiency and reduces heat losses. Additionally, by decreasing the power dissipation in these components, the need to increase the number of current drivers or to oversize them to handle higher-than-nominal power levels is reduced. This ultimately lowers the cost of lighting and signaling systems. Furthermore, this improvement directly impacts the lifespan of the battery powering the DC-DC converter, as well as the amount of CO_2_ generated, both of which benefit from the thermal adaptation proposed here.

For the case studies analyzed in this work, it was demonstrated that, with an optimized configuration in terms of resistor values and thermistor model selection, it was possible to achieve a relative reduction of 75% in the power dissipated by the current driver, as well as an improvement of more than 50% in the system’s relative efficiency for an LED branch.

## Figures and Tables

**Table 1:** Specifications of the lamp used.

Parameter	Module 1	Module 2	Module 3
Number of branches (*n*)	12	6	6
LED/branch (*m*)	2	2	2
LED model	DRS MKS	DRS MKS	DRA HKS
Current	13 mA	9 mA	30 mA
DC-DC model	MPS MPQ4322GLE		
Driver current model	E522.59	E522.59	E522.59

**Table 2:** Values used for the feedback loop of the DC-DC converter.

Configuration	R_0_ (kΩ)	B	R_11_ (kΩ)	R_12_ (kΩ)	R_2_ (kΩ)
#1	∞	∞	∞	43	4.75
#2	10	3380	200	43	4.3
#3	10	3380	13	2.1	0.23
#4	100	4250	77	7.2	0.815

## Data Availability

Data available on request due to restrictions.
